# The efficacy of transarterial chemoembolization combined with helical iodine-125 seed implant, lenvatinib and PD-1 inhibitors in patients with hepatocellular carcinoma complicated by main portal vein tumor thrombus: a retrospective study

**DOI:** 10.3389/fonc.2025.1514375

**Published:** 2025-05-08

**Authors:** Jia-Wen Lin, Shen Zhang, Jian Shen, Yu Yin, Jun Yang, Cai-Fang Ni, Wan-Sheng Wang

**Affiliations:** ^1^ Department of International Radiology, The First Affiliated Hospital of Soochow University, Suzhou, China; ^2^ Department of International Radiology, Zhongshan People’s Hospital, Zhongshan, China; ^3^ Department of International Radiology, The First People’s Hosiptal of Kunshan, Suzhou, China

**Keywords:** hepatocellular carcinoma, transarterial chemoembolization, helical iodine-125 seed implant, lenvatinib, programmed cell death ligand-1

## Abstract

**Purpose:**

To evaluate the efficacy and safety of a multimodal therapeutic approach involving transarterial chemoembolization (TACE) in conjunction with helical iodine-125 (I-125) seed implant, lenvatinib, and programmed cell death-1(PD-1) inhibitors for hepatocellular carcinoma (HCC) complicated by main portal vein tumor thrombus (MPVTT).

**Material and methods:**

HCC patients with MPVTT treated with TACE coupled with helical I-125 implant, lenvatinib, PD-1 inhibitors between September 2019 and August 2022 were retrospectively analyzed, and constituted as study group. Those treated with TACE, helical I-125 seed implant, and sorafenib between December 2016 and August 2020 served as the historical control group. All patients received sorafenib or lenvatinib combined with PD-1 inhibitors within 3–7 days after TACE and helical I-125 seed implantation. The longest follow-up period for all patients in both groups was 36 months from the date of helical I-125 seed implantation. Primary outcome was overall survival time (OS), and secondary outcomes were progression free survival time (PFS), objective response rate (ORR), and disease control rate (DCR). The Cox proportional hazards regression model was employed to identify independent prognostic factors influencing OS and PFS. The value *P* < 0.05 was deemed statistically significant.

**Results:**

A total of 53 patients were enrolled, with 22 assigned to the study group and 31 to the control group. The study group exhibited superior overall ORR(54.5% vs. 25.8%, *P* = 0.033) and overall DCR (77.3% vs. 64.5%, *P* = 0.319). Notably, the ORR and DCR of MPVTT were higher in the study group (86.4% vs. 51.6%, *P* = 0.008; and 95.5% vs. 83.9%, *P* = 0.382, respectively). Median OS (16.1 ± 6.1 months vs. 10.2 ± 0.8 months, *P* = 0.008) and PFS (13.6 ± 3.0 months vs. 6.1 ± 0.6 months, *P* = 0.014) were prolonged in the study group. The maximal tumor size, alpha fetoprotein level, and treatment modality were independent predictors for OS, while the maximal tumor size and treatment modality were independent determinants for PFS. Study group showed frequent hypothyroidism and reactive cutaneouscapillary (*P* < 0.01), with comparable grade 3/4 adverse events between groups.

**Conclusions:**

The integration of the helical I-125 seed implant with TACE, lenvatinib, and PD-1 inhibitors is the safe and efficacious approach in the management of HCC complicated by MPVTT.

## Introduction

Hepatocellular carcinoma (HCC) complicated by portal vein tumor thrombus (PVTT) is classified as Barcelona Clinic Liver Cancer (BCLC) stage C disease, where systemic therapies are recommended as the first-line treatment (1). Recent advancements in systemic therapies have yielded promising median overall survival (OS) of almost 2 years for HCC in BCLC stage C (2, 3). However, for patients with HCC complicated by main PVTT(MPVTT), the prognosis remains dismal. A *post hoc* analysis of the IMbrave150 study revealed that the median OS for such patients was merely 7.6 months and 5.5 months, respectively, treated with atezolizumab plus bevacizumab group(T+A) and the sorafenib (4).

The combined approach of endovascular brachytherapy with iodine-125 (I-125) seeds including I-125 seed strand plus stent (I-125 seed-stent), irradiation stent with I-125 seed and helical I-125 seed implant, and transarterial chemoembolization (TACE) is routinely utilized for the management of HCC complicated by MPVTT in China, with a favorable median OS ranging from 8.4 to 12.5 months (5–7). Furthermore, the incorporation of systemic therapies into endovascular brachytherapy with I-125 seeds and TACE has been shown to further enhance the prognostic outlook. Zhang et al. reported that a comprehensive treatment strategy incorporating I-125 seed strand-stent, TACE, molecular targeted therapies, and immune checkpoint inhibitors (ICIs) has remarkably extended the median OS of HCC with PVTT to 17.7 months (8).

Our previous researches demonstrated the safety and efficacy of integrating helical I-125 seed implant with TACE in the management of HCC with MPVTT (9). Juxtaposed against implantation of I-125 seed strand-stent or irradiation stent with I-125 seed, the helical I-125 seed implantation emerges as a less invasive approach, entailing reduced technical complexity. Whereas, the efficacy of combining helical I-125 seed implant with TACE and systemic therapies remains elusive. Therefore, the present study aims to elucidate a more efficacious treatment paradigm by assessing the therapeutic outcomes and safety profiles of treating HCC with MPVTT using helical I-125 seed implant in conjunction with TACE, lenvatinib, and programmed cell death-1 (PD-1) inhibitors, against a historical control of helical I-125 seed implant combined with TACE and sorafenib.

## Materials and methods

### Study design and patients

The clinical data of patients with HCC complicated by MPVTT who underwent TACE in combination with helical I-125 seed implant, lenvatinib, and PD-1 inhibitors in the first affiliated hospital of Soochow university between September 2019 and August 2022 were retrospectively collected. This cohort constituted the study group. Given the scarcity of cases where TACE, helical I-125 seed implant, and sorafenib were administered concurrently during the same period as the study group, records of HCC with MPVTT that underwent this combined therapy between December 2016 and August 2020 were gathered to form the historical control group. All patients underwent a standardized protocol involving synchronized helical I-125 seed implant and initial TACE treatment, followed by the introduction of a combination therapy with sorafenib, or lenvatinib plus PD-1 inhibitors, within 3 to 7 days. The inclusion criteria were as follows: (1) histological or radiological diagnosis of HCC based on the American Association for the Study of Liver Disease guideline; (2) HCC with Cheng’s type III PVTT; (3) at least one measurable tumor lesion in liver; (4) Child-Pugh class A/B with Eastern Cooperative Oncology Group performance status(ECOG-PS) of 0-2; (5) no prior history of systemic therapies; and (6) age range of 18–75 years, with life expectancy exceeding 3 months. Patients were excluded if they had: (1) tumor volume occupying 50% or more of the liver; (2) severe ascites, hepatic encephalopathy, or gastric fundal variceal bleeding; (3) high-flow intrahepatic arterioportal fistula; (5) coexistence of other malignancies; (6) brain metastasis; and (7) concurrent severe diseases affecting vital organs such as the heart, lungs, or kidneys. Informed consent was obtained from all patients participating in this retrospective study. This retrospective study conformed to the rules of the Declaration of Helsinki and was approved by the institutional review board. The requirement to obtain informed consent was waived.

### Treatment protocols

**Figure 1:**
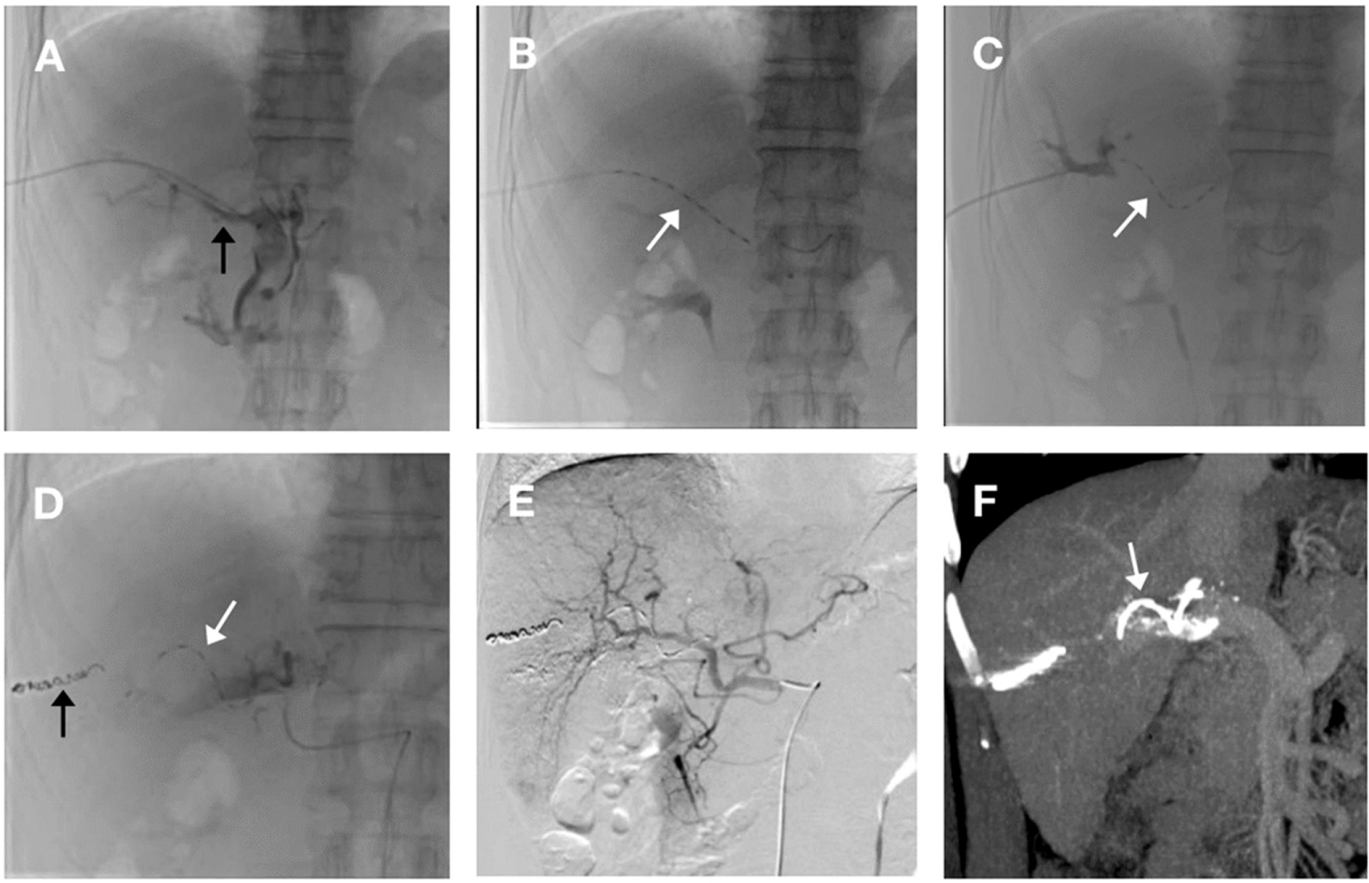
The procedure for helical Iodine-125 (I-125) seed implant and transarterial chemoembolization (TACE). **(A)** A 4-F catheter sheath was percutaneously inserted via transhepatic portal vein puncture into the right branch, subsequently advancing into the main portal vein. Portal venography reveals a filling defect in the main trunk, indicative of a main portal vein tumor thrombus (MPVTT) (black arrow). **(B)** Deployment of the helical I-125 seed was initiated through the catheter sheath. Prior to release, the seed was fully extended within the sheath(white arrow). **(C)** Following complete deployment, portal venography of the right branch demonstrated the helical I-125 seed self-anchoring in a spiral configuration within the main portal vein(white arrow). **(D)** After implantation of helical I-125 seed (white arrow), sealing of puncture tract using coil (black arrow). **(E)** Hepatic artery angiography after TACE treatment showed disappearance of staining in left liver tumor. **(F)** Enhanced computed tomography with portal vein phase reconstruction, performed after helical I-125 seed placement, clearly depicts the seed (white arrow) residing within the main portal vein, spanning across both proximal and distal extremities of the MPVTT.

The helical I-125 seed implant was composed of helical sleeves and I-125 seed. Production of the helical I-125 seed implant and the formula for calculating seed number were previously described in detail (7). An ultrasound-guided 22-gauge Chiba needle was used to puncture the left or right portal vein. A micro-guidewire was then introduced into the portal vein, a procedure which was confirmed by the injection of a contrast agent. Subsequently, a stiff guidewire was substituted. Thereafter, a 55-cm 4F sheath (Cook, US) was delivered along the guidewire to the portal vein, and the distal end of the main tumor thrombus was reached by the tip of the sheath. The helical I-125 seed implant, was loaded into the sheath and deployed in the portal vein at the site of MPVTT by pulling back the sheath to provide comprehensive coverage of the entire tumor thrombus. Following the deployment of the implant, the intrahepatic puncture tract was embolized with coils (**Figures 1A-D**). TACE was instantaneously performed following helical I-125 seed implantation. Following angiography, a solution consisting of 5–20 ml of lipiodol combined with pirarubicin was injected into the tumor feeding arteries via microcatheter. Subsequently, gelatin sponge particles with a diameter of 350-560μm were injected for embolization until the flow stasis of the tumor-feeding arterials was achieved under fluoroscopic guidance (**Figure 1E**). TACE was performed on demand in the event of intrahepatic tumor recurrence, the emergency of new tumors or the residual tumor lesions, following the rigorous exclusion of any contraindications. All of the treatment were performed by two interventionalists with more than 15-year experience.

The postoperative dose verification utilizing the Treatment Planning System (TPS) was rigorously executed via the 3D radiation treatment planning software, FtzyPlan version 1.3.118(Beijing FTT Technology Ltd., China), to precisely assess the radiation dose imparted to the brachytherapy target region following the helical I-125 seed implantation. Within the TPS framework, dedicated tools were employed to delineate the tumor thrombus contours and determine optimal particle implant locations and quantities, facilitating the generation of a concise dose-volume histogram, alongside an accurate calculation of the 90% target volume dose.

All patients adhered to the prescribed dosage of either lenvatinib (8mg/day for patients ≤60kg, 12mg/day for >60kg) or sorafenib (400mg administered twice daily) as molecular targeted therapies after and helical I-125 seed implantation and initial TACE. The dosage reduction or drug discontinuation was made in a timely manner based on the patient toleration. In addition, the two kinds of PD-1 inhibitors, namely sindilizumab and carelizumab, were administered in patients receiving lenvatinib after the first interventional therapy and repeated injection every 3 weeks. The drug dosage reduction, or, if necessary, complete drug discontinuation was made in a timely manner based on the onset of intolerance to adverse events.

### Follow-up and outcomes

The patients were subjected to contrast-enhanced computed tomography or magnetic resonance imaging scans every 6–8 weeks following interventional therapy, complicated by assessments of liver and kidney function, as well as routine blood tests. The primary endpoint was OS, measured from the initiation of interventional therapy to death or the final follow-up on December 31, 2022. To balance the follow-up duration between the two groups, the maximum follow-up duration for all patients in both groups was capped at 36 months after the helical I-125 seed implantation and initial TACE. The secondary endpoints included progression-free survival (PFS), the tumor response rate, and the treatment safety. Tumor response was evaluated adhering to modified Response Evaluation Criteria in Solid Tumors (RECIST), with a particular focus on MPVTT response, which was based on alterations in the maximum width of MPVTT. Specifically, complete response was defined as the absence of arterial phase enhancement or the complete disappearance of MPVTT; partial response (PR) entailed ≥ 30% reduction in MPVTT width from baseline; stable disease excluded both progressive disease (PD) and PR criteria; and PD was defined as ≥ 20% increase in MPVTT width or the extension of the thrombus to additional branches. Adverse events were graded according to the Common Terminology Criteria for Adverse Events version 5.0.

### Statistical analysis

Continuous variables were expressed as mean ± standard deviation, and Student’s t-test or Mann-Whitney U test were employed for between-group comparisons. The data of frequencies were analyzed by the chi-square test or Fisher’s exact test. OS and PFS was conducted using the Kaplan-Meier method and the log-rank test. Cox proportional hazards regression model was applied for univariate and multivariate analysis to ascertain the independent factors influencing the survival prognosis of HCC patients with MPVTT. Variables with *P* < 0.1 in the univariate analysis were included in the multivariate analysis. *P* < 0.05 was deemed statistically significant. All statistical analyses were conducted using SPSS 22.0 software (IBM, USA).

## Results

### Patients

A total of 53 patients were enrolled in the study, with 22 and 31 patients, respectively, in the study group and control group. The majority of patients(42/53) with HCC had a hepatitis B surface antigen-positive status. Most patients exhibited well-preserved liver function and a favorable ECOG-PS. Notably, 62.2%(33/53) of patients suffered from a maximal tumor size of 7 cm or greater, and approximately a quarter (14/53) complicated with extrahepatic metastasis. The baseline characteristics were comparable between the two groups (**Table 1**). Each patient successfully underwent a helical I-125 seed implant and result in self-fixation of the particles in a spiral configuration within the main portal vein. No displacement was observed in either group (**Figure 1F**). The study group and control group received radiation doses of 51.2 ± 5.4Gy and 53.9 ± 4.5Gy, respectively, for MPVTT, with no significant difference (*P* = 0.367). The average number of TACE procedures performed was 4.1 ± 1.8 in the study group and 3.6 ± 2.3 in control group (*P* = 0.446).

**Table 1:** Baseline characteristics of two groups.

Characteristics	Study group (n=22)	Control group (n=31)	*P*
Age, <55/≥55	12/10	16/15	0.833
Sex, male/female	19/3	27/4	1.000
HBsAg, +/-	18/4	24/7	0.964
Child-Pugh, A/B	15/7	25/6	0.299
ECOG-PS, 0-1/2	13/9	17/14	0.758
Tumor number, single/multiple	6/16	12/19	0.386
Maximal tumor size(cm), <7/≥7	9/13	11/20	0.688
AFP(ng/mL), <400/≥400	5/17	9/22	0.608
Extrahepatic metastasis, yes/no	6/16	8/23	0.905
Previous treatment, yes/no	3/19	5/26	1.000

ECOG, Eastern Cooperative Oncology Group performance status; AFP, alpha fetoprotein.

### Efficacy

**Figure 2:**
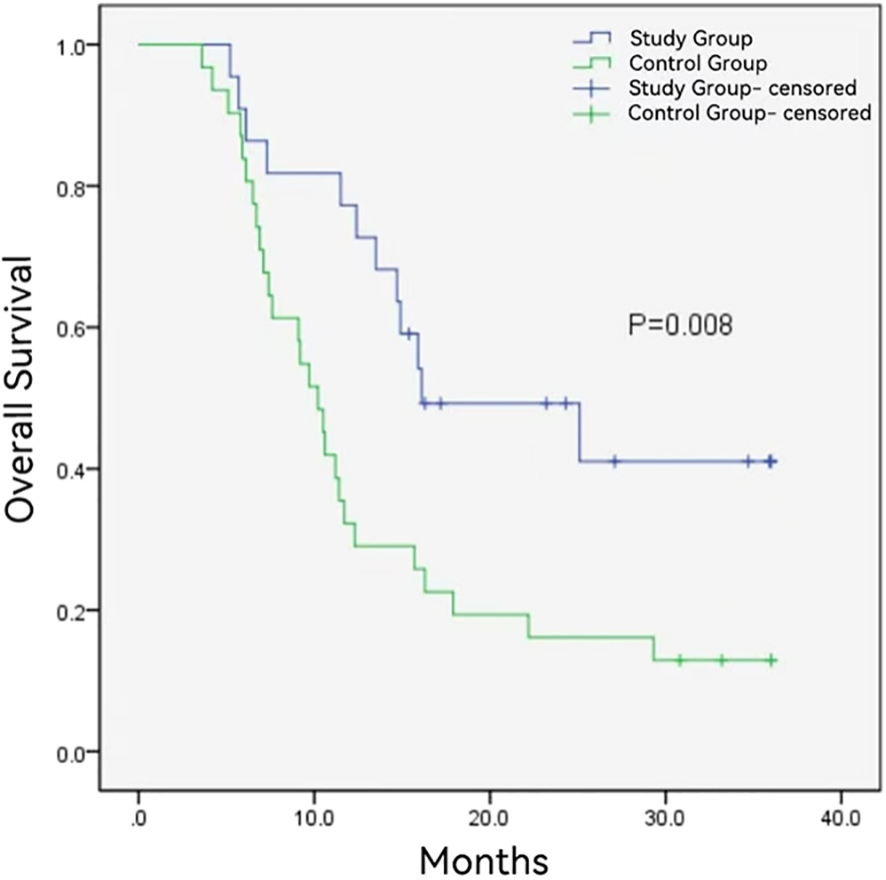
Kaplan-Meier analysis of overall survival in study group and control group.

**Figure 3:**
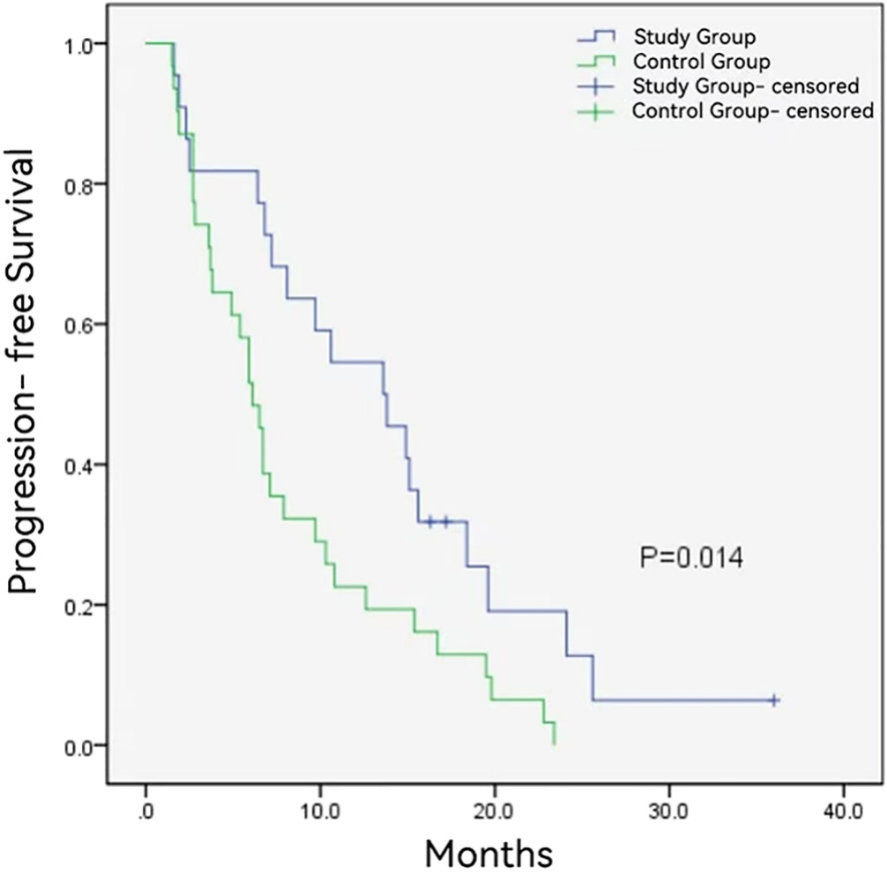
Kaplan-Meier analysis of progression free survival in study group and control group.

**Figure 4:**
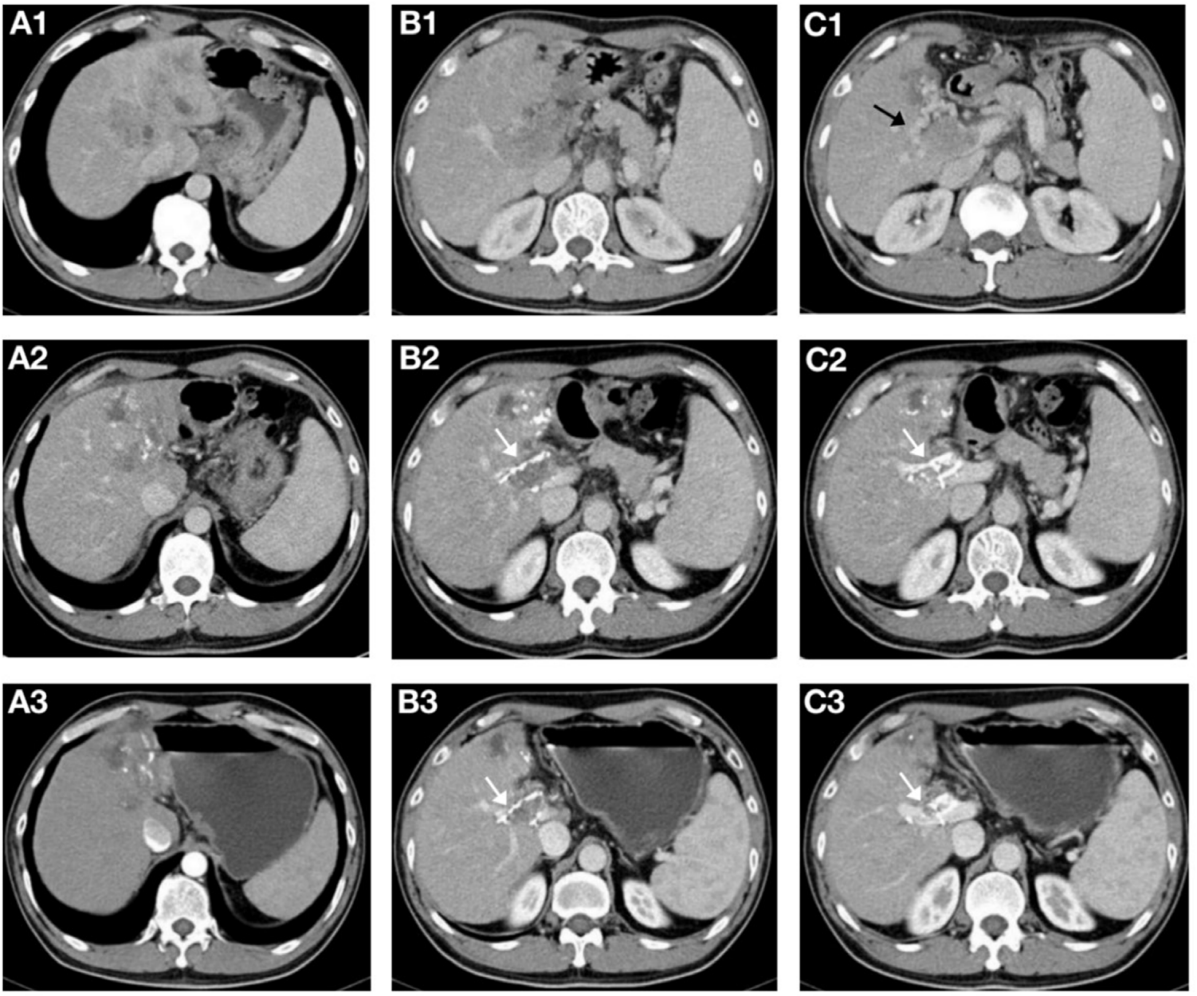
A 56-year-old male patient with hepatocellular carcinoma complicated with main portal vein tumor thrombus(MPVTT) treated with helical Iodine-125(I-125) seed implant, transarterial chemoembolization, lenvatinib, and PD-1 inhibitor. **(A1-C1)** Prior to the insertion of helical I-125 seed implant, the main trunk of the portal vein was occluded, forming peripheral collateral circulation(black arrows). **(A2-C2)** Two months after the insertion of the helical I-125 seed implant, the MPVTT and intrahepatic lesions shrank, and the peripheral collateral circulation of the main trunk of the portal vein disappeared; the helical I-125 seed implant can be seen beside the PVTT(white arrow). **(A3-C3)** After four months, the MPVTT and intrahepatic lesions further shrank, both achieving partial response, partial recanalization of the main trunk of the portal vein can be seen, and the helical I-125 seed implant did not shift (white arrow).

The follow-up duration in the control group and study group ranged from 3.6 to 36 months and 5.2 to 36 months, with a median follow-up time of 10.2 months and 16.0 months, respectively. During the follow-up period, 12 (54.5%) and 27 (87.1%) patients died in the study group and control group. The median OS was longer in the study group (16.1 ± 6.1months vs. 10.2 ± 0.8 months, *P* = 0.008) (**Figure 2**). Additionally, the study group demonstrated a favorable median PFS (13.6 ± 6.0 months vs. 6.1 ± 0.6, *P* = 0.014) (**Figure 3**), and a superior objective response rate (ORR) and disease control rate (DCR) for MPVTT compared to the control group(86.4% vs. 51.6%, *P* = 0.008; 95.5% vs. 83.9%, *P* = 0.382; respectively). Likewise, study group exhibited improved overall tumor control rates, with a favorable overall ORR (54.5% vs. 25.8%, *P* = 0.033) and overall DCR (77.3% vs. 64.5%, *P* = 0.319)(**Figure 4**). It is noteworthy that the two kinds PD-1 inhibitor employed in the study group (camrelizumab vs. sintilimab) exhibited comparable prognoses profiles, with no statistically significant differences observed in either median OS (16.9 ± 7.2 months vs. NA, *P* = 0.837) or median PFS (14.7 ± 4.9 vs. 13.9 ± 6.6 months, *P* = 0.756). In multivariate analysis, the maximal tumor size, alpha fetoprotein level, and treatment modality emerged as significant prognostic factors for OS (*P* all < 0.05)(**Table 2**). Furthermore, the maximal tumor size and treatment modality were independent prognosis for PFS (*P* all < 0.05) (**Table 3**).

**Table 2:** Univariate and multivariate analysis of OS in HCC with MPVTT.

Viable	Univariate analysis *P* HR (95% CI)	Multivariate analysis *P* HR (95% CI)
Ages, <55/≥55	0.781 0.914 (0.487-1.717)	
Gender, male/female	0.547 1.335 (0.521-3.419)	
HBsAg, +/-	0.469 0.759 (0.360-1.601)	
Child-Pugh, A/B	0.626 0.835 (0.405-1.723)	
ECOG-PS, 0-1/2	0.594 0.842 (0.447-1.584)	
Tumor number, single/multiple	0.723 0.886 (0.454-1.731)	
Maximal tumor size(cm), <7/≥7	0.020 0.445 (0.224-0.882)	0.039 0.472 (0.231-0.962)
AFP (ng/mL), <400/≥400	0.047 0.431 (0.188-0.991)	0.028 0.371 (0.153-0.898)
Extrahepatic spread, yes/no	0.292 0.686 (0.340-1.383)	
Previous treatment, yes/no	0.864 0.927 (0.387-2.221)	
Treatment, study/control	0.010 0.407 (0.205-0.810)	0.001 0.293 (0.143-0.599)

OS, overall survival; HCC, hepatocellular carcinoma; MPVTT, main portal vein tumor thrombus; ECOG-PS, Eastern Cooperative Oncology Group performance status; AFP, alpha fetoprotein.

**Table 3:** Univariate and multivariate analysis of PFS in HCC with MPVTT.

Viable	Univariate analysis *P* HR (95% CI)	Multivariate analysis *P* HR (95% CI)
Ages, <55/≥55	0.244 1.421 (0.787-2.564)	
Gender, male/female	0.392 1.458 (0.615-3.460)	
HBsAg, +/-	0.341 0.709 (0.349-1.439)	
Child-Pugh, A/B	0.764 0.905 (0.471-1.739)	
ECOG-PS, 0-1/2	0.736 1.102 (0.627-1.939)	
Tumor number, single/multiple	0.622 0.861 (0.476-1.559)	
Maximal tumor size(cm), <7/≥7	0.011 0.466 (0.258-0.842)	0.001 0.346 (0.183-0.653)
AFP (ng/mL), <400/≥400	0.329 0.725 (0.380-1.382)	
Extrahepatic spread, yes/no	0.931 0.972 (0.506-1.867)	
Previous treatment, yes/no	0.521 0.767 (0.342-1.721)	
Treatment, study/control	0.017 0.482 (0.265-0.878)	0.002 0.353 (0.185-0.672)

OS, overall survival; HCC, hepatocellular carcinoma; MPVTT, main portal vein tumor thrombus; ECOG-PS, Eastern Cooperative Oncology Group performance status; AFP, alpha fetoprotein.

### Safety

In the study group, the predominant adverse events(AEs) encompassed fever, elevated aminotransferases, abdominal pain, and reactive cutaneouscapillary(RCCEP). Conversely, the control group exhibited a high frequency of hand-foot skin reaction, fever, elevated aminotransferases, and diarrhea as AEs. Notably, the hypothyroidism and RCCEP were frequently witnessed in patients in study group(*P* all < 0.01). This may be associated with PD-1 inhibitor. In the study group, all patients who developed hypothyroidism and RCCEP were classified as Grade 1/2 AEs. These patients continued to receive the current PD-1 therapy while being managed with symptomatic supportive care. Any incidence of AEs in grade 3/4 were comparable between the two groups(*P* all >0.05)(**Table 4**), and no fatality attributed to AEs.

**Table 4:** AEs After Treatments.

AEs	Grade	Study group	Control group	
AST increase	Any grade	54.5% (10)	41.9% (11)	0.365
	Grade 3/4	0	3.2% (1)	1.000
ALT increase	Any grade	40.1% (9)	35.5% (12)	0.688
	Grade 3/4	0	3.2% (1)	1.000
Abdominal pain	Any grade	50.0% (12)	35.5% (12)	0.291
	Grade 3/4	0	3.2% (1)	1.000
Fever	Any grade	59.1% (11)	45.2% (13)	0.318
	Grade 3/4	0	0	–
Hyperbilirubinemia	Any grade	22.7% (5)	22.6% (7)	1.000
	Grade 3/4	0	0	–
Hypertension	Any grade	36.4% (8)	22.6% (7)	0.272
	Grade 3/4	18.2% (4)	9.6% (3)	0.625
Diarrhea	Any grade	36.4% (8)	40.1% (11)	0.683
	Grade 3/4	0	3.2% (1)	1.000
HFSR	Any grade	27.3% (6)	48.4% (14)	0.121
	Grade 3/4	4.5% (1)	9.6% (3)	1.000
Proteinuria	Any grade	27.3% (6)	9.6% (3)	0.190
	Grade 3/4	4.5% (1)	0	0.415
Fatigue	Any grade	31.8% (7)	25.8% (8)	0.632
	Grade 3/4	0	3.2% (1)	1.000
Vomiting	Any grade	18.2% (4)	22.6% (7)	0.964
	Grade 3/4	0	0	–
Nausea	Any grade	18.2% (4)	22.5% (7)	0.964
	Grade 3/4	0	0	–
Hoarseness	Any grade	22.7% (5)	6.4% (2)	0.189
	Grade 3/4	0	0	–
Autoimmune hepatitis	Any grade	4.5% (1)	0	0.415
	Grade 3/4	4.5% (1)	0	0.415
Hypothyroidism	Any grade	31.8% (7)	0	0.003
	Grade 3/4	0	0	–
RCCEP	Any grade	40.1% (9)	0	<0.001
	Grade 3/4	4.5% (1)	0	0.415

AEs, adverse events; AST, aspartate aminotransferase; ALT, alanine aminotransferase; HFSR, hand-foot skin reaction; RCCEP, reactive cutaneouscapillary endothelial proliferation.

## Discussion

This study utilized helical I-125 seed implantation, TACE and sorafenib as the historical control group to comparatively analyze the efficacy and safety of helical I-125 seed implantation combined with TACE, lenvatinib, and PD-1 inhibitors in the treatment of HCC with MPVTT. The results demonstrate that compared to the control group, the study group exhibits a better survival prognosis of HCC patients with MPVTT, with acceptable safety profiles for both treatment modalities. Notably, the discrepancy in follow-up duration between the two groups can be ascribed to inferior treatment outcomes, shorted survival, and a higher incidence of lost-to-follow-up cases in patients in control group.

Though BCLC stage system recommends systemic therapy for advanced HCC, systemic therapy has shown limited efficacy in patients with PVTT, particularly in cases involving the MPVTT (1, 4). I-125 seed-stent, irradiation stent with I-125 seed, and helical I-125 seed implantation have been reported as treatment modalities of endovascular brachytherapy with I-125 seeds to the management of HCC with MPVTT (5–7). The fundamental premise behind employing I-125 seed strand-stent or irradiation stent with I-125 seed for MPVTT is to facilitate recanalization the main portal vein, rejuvenating hepatic blood flow, and thereby mitigating liver dysfunction stemming from insufficient compensatory perfusion via an obstructed or stenotic portal vein after TACE-induced hepatic artery occlusion (6, 15). Whereas, many recent investigations have conclusively demonstrated the safety and efficacy of super-selective TACE or TACE-based combination therapies in the management of HCC with MPVTT, without the necessity for portal vein stenting (10–13). Consequently, the recanalization of the stenotic or obstructed portal vein is not mandatory when combining I-125 seed endovascular brachytherapy with TACE for the treatment of HCC in the presence of MPVTT. Our prior research underscores that, irrespective of portal vein stenosis severity, the helical I-125 seed implant can autonomously adhere adjacent to MPVTT, inhibiting its progression, without reliance on stent compression (7). This approach, coupled with TACE, exhibits favorable safety and efficacy profiles in the management of HCC with MPVTT. In comparison with the use of I-125 seed stent that is introduced through a 10-French sheath and necessitate anticoagulation therapy, helical I-125 seed implantation has been shown to be a less invasive procedure, less technically complex, and does not require anticoagulation therapy (14). Moreover, in comparison with I-125 seed strand, which has a risk of displacement, helical I-125 seed implantation has been demonstrated to be self-retaining (7).

Zhang et al. documented that I-125 seed strand-stent combined with TACE and sorafenib significantly elongated median OS (10.3 months vs. 6.0 months, *P* < 0.001), time to progression (9.0 months vs. 3.4 months, *P* < 0.001), ORR (45.9% vs. 16.1%, *P* = 0.009) and DCR (67.6% vs. 29.0%, *P* = 0.002) in HCC with MPVTT, as compared to TACE plus sorafenib. Furthermore, the safety of minimally invasive implantation of I-125 seeds strand-stent through a 6-F sheath was demonstrated (16). Conversely, an enlarged sheath is associated with the potential for increased complications, though these may be manageable. In a multicenter randomized controlled trial conducted by Lu et al., the introduction of an irradiation stent with I-125 through a 10-F sheath has been shown to induce more frequent abdominal pain(29% vs. 12%) though the combination of an irradiation stent with I-125 seeds with TACE has yielded a superior median OS (9.9 months vs. 6.3 months, *P* = 0.01), favorable tumor complete rate(21.6% vs. 5.9%, *P* = 0.109) and DCR(86.0% vs. 67.0%, *P* = 0.022) compared to sorafenib plus TACE in patients with HCC complicated by MPVTT (14). Notably, the historical control arm in this study achieved a commendable OS of 10.2 months, complicated by a satisfactory ORR(51.6%) and DCR(83.9%) for MPVTT. Despite the BCLC continuing to advocate T+A therapy as the preferred treatment for advanced HCC, the effectiveness of this approach for Vp4 HCC remains restricted (OS, 7 months; PFS, 5.4 months; and ORR, 23%) (4). Concurrently, patients undergoing T+A therapy exhibited an elevated risk of bleeding complication. The aforementioned treatment strategies that combined I-125 seeds endovascular brachytherapy with TACE or sorafenib have demonstrated a better prognosis profile (4, 6, 16). This may be attributed to the effective inhibition of multipolar vascular tumor thrombus by endovascular brachytherapy using I-125 seeds, thereby broadening the therapeutic spectrum and optimizing the therapeutic window for both TACE and systemic therapies. Nonetheless, T+A currently represents the highest level of evidence-based therapy for advanced HCC. The combination of TACE, helical I-125 seed implant, and T+A may offer enhanced therapeutic potential. This multimodal approach has the potential to leverage the ability of I-125 irradiation to recanalize portal vein thrombosis and mitigate bleeding risk.

In recent years, there has been an increasing focus on the field of tumor immunoregulation and therapy. It is imperative to acknowledge the intricate interplay between local and systemic therapy, particularly in the context of immune regulation. The immunosuppressive state of HCC itself is the primary factor hindering the efficacy of ICIs monotherapy, which is unable to significantly improve tumor response (17, 18). Radiotherapy and locoregional therapies, such as TACE, are postulated to induce immunogenic tumor necrosis, transforming the tumor from a ‘cold’ to a ‘hot’ state. Consequently, this transformation is anticipated to ultimately improve the tumor immune microenvironment(TIME) and present an avenue for the administration of ICIs (19). It has been demonstrated that TACE can upregulate PD-L1 expression, thus creating a favorable environment for subsequent anti-PD-1 or anti-PD-L1 monoclonal antibody therapy (20). However, it has also been reported that embolization may lead to a reduction in CD8^+^T cell infiltration, which might necessitate molecular targeted agents to recruit CD8^+^T cells (21). Lenvatinib, an effective anti-angiogenic agent, has the potential to improve the TIME in HCC by inhibiting the differentiation and stability of Tregs by targeting FGFR4 (22, 23). It is hypothesized that the administration of lenvatinib prior to and following TACE results in a reduction in tumor interstitial osmotic pressure by normalizing tumor blood vessels and reducing microvascular density. This, in turn, has been believed to increase drug delivery to maximize the efficacy of TACE (24). Furthermore, the use of anti-angiogenic agents has been shown to enhance the efficacy of ICIs. In HCC murine models, the combination of lenvatinib and PD-1 inhibitors demonstrated a superior anti-tumor efficacy over monotherapy. Additionally, lenvatinib reduces monocyte and macrophage populations while, in conjunction with PD-1 inhibitors, enhances the proportion of activated CD8^+^T cells (25). The Phase Ib studies of lenvatinib combined with pembrolizumab yielded promising anti-tumor responses, and the subsequent LEAP-012 trial further confirmed the therapeutic benefits of TACE in combination with lenvatinib and pembrolizumab (26, 27). Recently, Zhang et al. reported the safety and efficacy of augmenting PD-1 inhibitors to I-125 seed strand-stent, TACE, and lenvatinib in HCC with MPVTT, achieving a significantly prolonged median OS(17.7 months vs. 12.0 months, *P* = 0.01) (8). This finding underlines the synergistic anti-tumor effects of combining lenvatinib and PD-1 inhibitors with locoregional treatments, thereby enhancing survival prognosis. In the present study, compared with helical I-125 seed implant with TACE and sorafenib, the utilization of helical I-125 seed implant in conjunction with TACE, lenvatinib, and PD-1 inhibitors for HCC with MPVTT extend median OS to 16.1 months, mirroring the OS of 17.7 months by Zhang et al. and surpassing previous systemic treatment outcomes (8). The combination of radiotherapy with anti-angiogenic agents and PD-L1 monoclonal antibodies has been shown to activate exhausted T lymphocytes (28). In addition, the impact of radiotherapy and brachytherapy on the TIME, including changes in immune cells and cytokines, is also under investigation (29, 30). In a Phase II study, patients with HCC and macrovascular invasion experienced a 38.0% improvement in ORR after radiotherapy with a PD-1 monoclonal antibody compared to radiotherapy alone (31). From these perspectives, the combination of TACE, I-125 seeds brachytherapy, and lenvatinib may induce more profound improvements or alterations in the TIME. However, further in-depth basic research is required to ascertain whether this combination exerts a cascading amplification effect on immune activation.

This study is inherently constrained by several limitations. Firstly, its retrospective nature predisposes it to various confounding biases, which may compromise the robustness of the findings. Furthermore, owing to the absence of contemporaneous control cases involving TACE in conjunction with helical I-125 seed implant and sorafenib, a historical control group is devised as an alternative. Lastly, the modest sample sizes within both the study and the historical control cohorts pose challenges, and the employment of two distinct PD-1 inhibitors(camrelizumab and sintilimab) introduces a potential variable that may influence the precision of the outcomes. Multicenter prospective study is wanted to born out the present findings.

## Conclusion

The integration of the helical I-125 seed implant with TACE, lenvatinib, and PD-1 inhibitors in the management of HCC concurrent with MPVTT demonstrates a safe and efficacious therapeutic approach.

## Data Availability

The datasets generated and/or analyzed during the current study are contained within the Office of Department of Interventional Radiology, the First Affiliated Hospital of Soochow University but are not publicly available because confidentiality, security and ownership matters. They may be available from the corresponding author upon reasonable request.
